# (*Z*)-1,4-Diphenyl­but-1-en-3-ynyl acetate

**DOI:** 10.1107/S1600536812037439

**Published:** 2012-09-05

**Authors:** Zheng-Wang Chen, Hai-Chuan Chen, Dong-Nai Ye, Qiao-Sheng Hu

**Affiliations:** a Key Laboratory of Organo-Pharmaceutical Chemistry of Jiangxi Province, Gannan Normal University, Gan Zhou 341000, People’s Republic of China

## Abstract

The title compound, C_18_H_14_O_2_, is almost planar with a dihedral angle of 1.24 (2)° between the phenyl­ethynyl and styryl groups. The acet­oxy group is tilted by 82.46 (2) and 82.26 (3)° with respect to the benzene ring planes.

## Related literature

For general background to title compound, see: Goossen & Paetzold (2004 ▶); Debergh *et al.* (2008 ▶); Li *et al.* (2010 ▶); Nakao *et al.* (2008 ▶); Chen *et al.* (2011 ▶). For bond-length data, see: Allen *et al.* (1987 ▶).
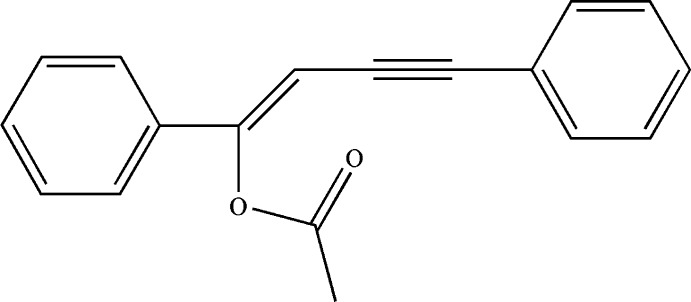


## Experimental

### 

#### Crystal data

C_18_H_14_O_2_
*M* *_r_* = 262.29
Monoclinic, 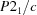

*a* = 13.1480 (5) Å
*b* = 5.5912 (2) Å
*c* = 19.7579 (7) Å
β = 91.558 (2)°
*V* = 1451.93 (9) Å^3^
*Z* = 4
Mo *K*α radiation
μ = 0.08 mm^−1^
*T* = 296 K
0.33 × 0.28 × 0.20 mm

#### Data collection

Bruker APEXII area-detector diffractometer
Absorption correction: multi-scan (*SADABS*; Sheldrick, 1996 ▶) *T*_min_ = 0.987, *T*_max_ = 0.998
8119 measured reflections
2611 independent reflections
1678 reflections with *I* > 2σ(*I*)
*R*_int_ = 0.021

#### Refinement

*R*[*F* ^2^ > 2σ(*F* ^2^)] = 0.041
*wR*(*F* ^2^) = 0.123
*S* = 1.02
2611 reflections
182 parameters
H-atom parameters constrained
Δρ_max_ = 0.09 e Å^−3^
Δρ_min_ = −0.14 e Å^−3^

### 

Data collection: *APEX2* (Bruker, 2004 ▶); cell refinement: *SAINT* (Bruker, 2004 ▶); data reduction: *SAINT*; program(s) used to solve structure: *SHELXS97* (Sheldrick, 2008 ▶); program(s) used to refine structure: *SHELXL97* (Sheldrick, 2008 ▶); molecular graphics: *XP* in *SHELXTL* (Sheldrick, 2008 ▶); software used to prepare material for publication: *SHELXL97*.

## Supplementary Material

Crystal structure: contains datablock(s) I, global. DOI: 10.1107/S1600536812037439/hg5238sup1.cif

Structure factors: contains datablock(s) I. DOI: 10.1107/S1600536812037439/hg5238Isup2.hkl

Supplementary material file. DOI: 10.1107/S1600536812037439/hg5238Isup3.cml

Additional supplementary materials:  crystallographic information; 3D view; checkCIF report

## Figures and Tables

**Fig. 1.**
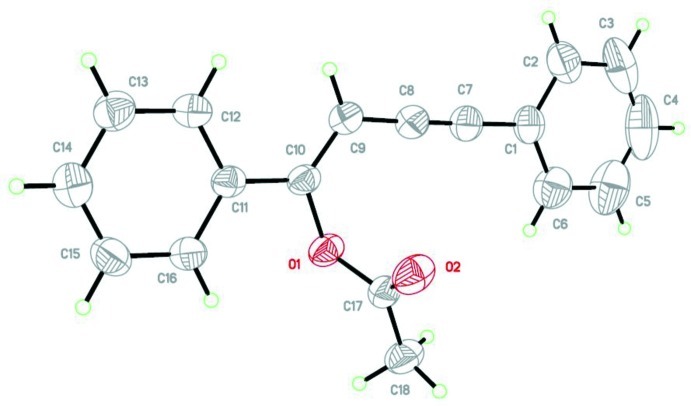
The molecular structure of the tile compound, showing 50% probability displacement ellipsoids and the atom-numbering scheme.

**Fig. 2.**
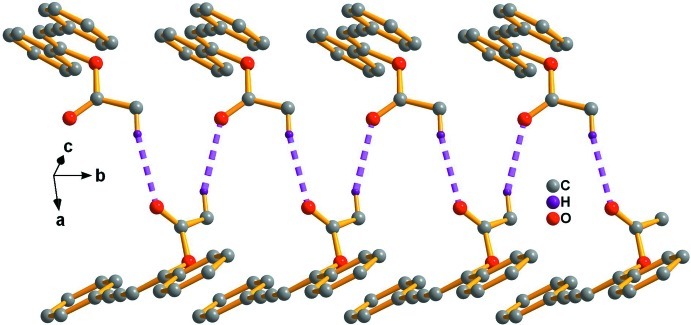
An infinite chain of the title compound; C—H···O interactions are shown as dashed lines. The H-atoms not involved in H-bonds have been excluded for clarity.

## supplementary crystallographic information

### Comment

The title compound, (I), C_19_H_17_N_3_O_2_, is a multifunctional compound, which can achieve varieties of conversion. For example, enol acetates was frequently used as intermediates in organic synthesis and pharmaceutical chemistry (Goossen *et al.*, 2004; Debergh *et al.*, 2008), the enyne derivatives had high synthetic potential due to wide applicability (Li *et al.*, 2010; Nakao *et al.*, 2008), and the enyne acetate could be converted to heterocyclic compounds through metal-catalyzed transformation or electrophilic cyclization (Chen *et al.*, 2011). Moreover, the (*Z*)-enyne acetate was obtained from (*Z*)-2-bromoenol acetate and phenylacetylene, it proved that the Sonogashira coupling reaction was in stereospecific manner. In view of this, the crystal structure determination of the title compound was carried out and the results are presented here.

As depicted in Fig. 1, the phenylethynyl group (C1—C8) [maximum deviations of 0.007 (2) and 0.028 Å for the C7 and C8 atoms, respectively] and the styryl group (C9—C16) [maximum deviations of 0.058 (2) and 0.041 (3) Å for the C9 and C10 atoms, respectively] are almost planar with maximum deviation of 1.24 (2) °. The acetoxy group (C17/C18/O1/O2) is slight tilted with respect to the benzene mean planes by 82.46 (2) (C11—C16) and 82.26 (3) ° (C1—C6). The bond lengths are within normal range (Allen *et al.*, 1987). The molecules are linked into an infinite chain through intermolecular C18—H18A···O2 hydrogen bonding interactions. In addition, intramolecular C16—H16···O1 are also observed.

### Experimental

To the mixture of (*Z*)-2-bromoenol acetate (1 mmol, 0.241 g), Pd(OAc)_2_ (0.05 mmol, 0.011 g) and PPh_3_ (0.1 mmol, 0.026 g) in THF (2 ml) solvent, TEA (1 mmol, 0.101 g) and CuI (0.05 mmol, 0.0098 g) were added successively, stirred for five minutes at room temperature, phenylacetylene (2.0 mmol, 0.204 g) was added, the flask was then sealed and stirred at 323 K for 6 h. The solution was washed with water (10 ml) and extracted with ethyl acetate (24 ml), and the combined extract was dried with anhydrous MgSO_4_. Solvent was removed, and the residue was purified by silica gel (200–300 mesh) column by elution with petroleum ether: ethyl acetate (10:1) to give 20 fractions (200 ml per fraction). The title compound (252.8 mg) was isolated from the fractions 5–16 (yield 96.5%). Single crystals suitable for X-ray diffraction were prepared by slow evaporation of a solution of the title compound in ethyl acetate at room temperature.

### Refinement

All H atoms were located on the difference maps, and were treated as riding atoms with C—H distances of 0.96Å for methyl, with *U*_iso_(H) = 1.5*U*_eq_ (methyl C-atoms) and 1.2*U*_eq_(non-methyl C-atoms). The hightest peak is located 1.07Å from O2 and the deepest hole is located 0.97 Å from C6.

### Crystal data

**Table:**

C_18_H_14_O_2_	*F*(000) = 552
*M**_r_* = 262.29	*D*_x_ = 1.200 Mg m^−^^3^
Monoclinic, *P*2_1_/*c*	Mo *K*α radiation, λ = 0.71073 Å
Hall symbol: -P 2ybc	Cell parameters from 5837 reflections
*a* = 13.1480 (5) Å	θ = 2.8–27.9°
*b* = 5.5912 (2) Å	µ = 0.08 mm^−^^1^
*c* = 19.7579 (7) Å	*T* = 296 K
β = 91.558 (2)°	Block, colorless
*V* = 1451.93 (9) Å^3^	0.33 × 0.28 × 0.20 mm
*Z* = 4	

### Data collection

**Table:**

Bruker APEXII area-detector diffractometer	2611 independent reflections
Radiation source: fine-focus sealed tube	1678 reflections with *I* > 2σ(*I*)
Graphite monochromator	*R*_int_ = 0.021
φ and ω scan	θ_max_ = 25.2°, θ_min_ = 2.6°
Absorption correction: multi-scan (*SADABS*; Sheldrick, 1996)	*h* = −15→15
*T*_min_ = 0.987, *T*_max_ = 0.998	*k* = −6→6
8119 measured reflections	*l* = −23→23

### Refinement

**Table:**

Refinement on *F*^2^	Primary atom site location: structure-invariant direct methods
Least-squares matrix: full	Secondary atom site location: difference Fourier map
*R*[*F*^2^ > 2σ(*F*^2^)] = 0.041	Hydrogen site location: inferred from neighbouring sites
*wR*(*F*^2^) = 0.123	H-atom parameters constrained
*S* = 1.02	*w* = 1/[σ^2^(*F*_o_^2^) + (0.0583*P*)^2^ + 0.1384*P*] where *P* = (*F*_o_^2^ + 2*F*_c_^2^)/3
2611 reflections	(Δ/σ)_max_ < 0.001
182 parameters	Δρ_max_ = 0.09 e Å^−^^3^
0 restraints	Δρ_min_ = −0.14 e Å^−^^3^

### Special details

**Table:**

Geometry. All e.s.d.'s (except the e.s.d. in the dihedral angle between two l.s. planes) are estimated using the full covariance matrix. The cell e.s.d.'s are taken into account individually in the estimation of e.s.d.'s in distances, angles and torsion angles; correlations between e.s.d.'s in cell parameters are only used when they are defined by crystal symmetry. An approximate (isotropic) treatment of cell e.s.d.'s is used for estimating e.s.d.'s involving l.s. planes.
Refinement. Refinement of *F*^2^ against ALL reflections. The weighted *R*-factor *wR* and goodness of fit *S* are based on *F*^2^, conventional *R*-factors *R* are based on *F*, with *F* set to zero for negative *F*^2^. The threshold expression of *F*^2^ > σ(*F*^2^) is used only for calculating *R*-factors(gt) *etc*. and is not relevant to the choice of reflections for refinement. *R*-factors based on *F*^2^ are statistically about twice as large as those based on *F*, and *R*- factors based on ALL data will be even larger.

### Fractional atomic coordinates and isotropic or equivalent isotropic displacement parameters (Å^2^)

**Table:**

	*x*	*y*	*z*	*U*_iso_*/*U*_eq_	
C1	0.30322 (13)	−0.0799 (4)	0.49684 (9)	0.0692 (5)	
C2	0.28690 (17)	−0.2708 (5)	0.53970 (12)	0.0953 (7)	
H2	0.2419	−0.3916	0.5266	0.114*	
C3	0.3369 (3)	−0.2826 (7)	0.60151 (15)	0.1280 (11)	
H3	0.3254	−0.4111	0.6302	0.154*	
C4	0.4031 (3)	−0.1080 (9)	0.62109 (15)	0.1426 (16)	
H4	0.4372	−0.1181	0.6628	0.171*	
C5	0.4196 (2)	0.0823 (8)	0.57961 (17)	0.1327 (12)	
H5	0.4647	0.2021	0.5933	0.159*	
C6	0.36951 (17)	0.0975 (5)	0.51739 (11)	0.0974 (7)	
H6	0.3807	0.2280	0.4894	0.117*	
C7	0.25155 (13)	−0.0651 (4)	0.43211 (10)	0.0701 (5)	
C8	0.20928 (13)	−0.0556 (4)	0.37789 (10)	0.0685 (5)	
C9	0.15912 (12)	−0.0526 (4)	0.31331 (9)	0.0652 (5)	
H9	0.1158	−0.1790	0.3023	0.078*	
C10	0.17019 (11)	0.1197 (3)	0.26765 (8)	0.0550 (4)	
C11	0.11939 (11)	0.1374 (3)	0.20075 (8)	0.0536 (4)	
C12	0.04989 (12)	−0.0339 (3)	0.17850 (8)	0.0650 (5)	
H12	0.0366	−0.1650	0.2058	0.078*	
C13	0.00025 (14)	−0.0127 (4)	0.11645 (10)	0.0748 (5)	
H13	−0.0463	−0.1290	0.1024	0.090*	
C14	0.01901 (15)	0.1784 (4)	0.07548 (9)	0.0752 (6)	
H14	−0.0148	0.1929	0.0337	0.090*	
C15	0.08782 (15)	0.3482 (4)	0.09637 (10)	0.0799 (6)	
H15	0.1012	0.4776	0.0684	0.096*	
C16	0.13779 (13)	0.3297 (3)	0.15865 (10)	0.0708 (5)	
H16	0.1840	0.4472	0.1723	0.085*	
C17	0.33312 (11)	0.2969 (3)	0.28114 (8)	0.0565 (4)	
C18	0.38508 (13)	0.5103 (4)	0.30975 (10)	0.0770 (6)	
H18A	0.4559	0.5052	0.2992	0.116*	
H18B	0.3783	0.5121	0.3580	0.116*	
H18C	0.3548	0.6521	0.2906	0.116*	
O1	0.23034 (7)	0.3175 (2)	0.28623 (6)	0.0629 (3)	
O2	0.37147 (8)	0.1261 (2)	0.25663 (7)	0.0784 (4)	

### Atomic displacement parameters (Å^2^)

**Table:**

	*U*^11^	*U*^22^	*U*^33^	*U*^12^	*U*^13^	*U*^23^
C1	0.0638 (10)	0.0764 (14)	0.0674 (11)	0.0162 (10)	0.0031 (9)	−0.0050 (11)
C2	0.0945 (15)	0.0937 (18)	0.0979 (16)	0.0216 (13)	0.0047 (12)	0.0187 (14)
C3	0.146 (3)	0.151 (3)	0.0871 (19)	0.074 (2)	0.0098 (17)	0.0319 (19)
C4	0.136 (3)	0.212 (5)	0.0786 (19)	0.094 (3)	−0.0156 (18)	−0.027 (2)
C5	0.122 (2)	0.164 (3)	0.111 (2)	0.018 (2)	−0.0324 (18)	−0.054 (2)
C6	0.0986 (16)	0.1039 (19)	0.0890 (15)	−0.0034 (15)	−0.0102 (12)	−0.0148 (14)
C7	0.0626 (10)	0.0702 (14)	0.0776 (13)	0.0052 (9)	0.0032 (9)	0.0003 (10)
C8	0.0592 (10)	0.0669 (13)	0.0792 (12)	−0.0019 (9)	0.0016 (9)	0.0049 (10)
C9	0.0571 (9)	0.0617 (13)	0.0767 (12)	−0.0069 (9)	−0.0037 (8)	0.0016 (10)
C10	0.0426 (8)	0.0485 (11)	0.0743 (11)	−0.0014 (7)	0.0055 (7)	−0.0032 (9)
C11	0.0447 (8)	0.0499 (11)	0.0667 (10)	0.0022 (8)	0.0108 (7)	−0.0005 (8)
C12	0.0709 (11)	0.0572 (12)	0.0670 (11)	−0.0081 (9)	0.0066 (8)	0.0003 (9)
C13	0.0796 (12)	0.0722 (14)	0.0725 (12)	−0.0078 (11)	−0.0020 (9)	−0.0111 (11)
C14	0.0783 (12)	0.0820 (15)	0.0654 (11)	0.0095 (12)	0.0042 (9)	−0.0022 (11)
C15	0.0782 (12)	0.0777 (15)	0.0842 (13)	0.0033 (11)	0.0102 (10)	0.0242 (12)
C16	0.0594 (10)	0.0629 (13)	0.0900 (13)	−0.0070 (9)	0.0010 (9)	0.0126 (11)
C17	0.0472 (9)	0.0560 (11)	0.0664 (10)	0.0011 (8)	0.0024 (7)	0.0011 (9)
C18	0.0659 (11)	0.0718 (14)	0.0930 (13)	−0.0169 (10)	−0.0036 (9)	−0.0095 (11)
O1	0.0477 (6)	0.0512 (8)	0.0900 (8)	−0.0005 (5)	0.0040 (5)	−0.0097 (6)
O2	0.0554 (7)	0.0700 (9)	0.1103 (10)	0.0034 (6)	0.0143 (6)	−0.0190 (8)

### Geometric parameters (Å, º)

**Table:**

C1—C6	1.374 (3)	C11—C16	1.385 (2)
C1—C2	1.383 (3)	C11—C12	1.387 (2)
C1—C7	1.434 (2)	C12—C13	1.378 (2)
C2—C3	1.373 (4)	C12—H12	0.9300
C2—H2	0.9300	C13—C14	1.367 (3)
C3—C4	1.357 (5)	C13—H13	0.9300
C3—H3	0.9300	C14—C15	1.367 (3)
C4—C5	1.364 (5)	C14—H14	0.9300
C4—H4	0.9300	C15—C16	1.383 (2)
C5—C6	1.381 (3)	C15—H15	0.9300
C5—H5	0.9300	C16—H16	0.9300
C6—H6	0.9300	C17—O2	1.1894 (19)
C7—C8	1.194 (2)	C17—O1	1.3626 (18)
C8—C9	1.420 (2)	C17—C18	1.479 (2)
C9—C10	1.330 (2)	C18—H18A	0.9600
C9—H9	0.9300	C18—H18B	0.9600
C10—O1	1.4025 (18)	C18—H18C	0.9600
C10—C11	1.468 (2)		
			
C6—C1—C2	118.9 (2)	C16—C11—C10	120.70 (15)
C6—C1—C7	120.2 (2)	C12—C11—C10	121.23 (15)
C2—C1—C7	120.9 (2)	C13—C12—C11	120.95 (18)
C3—C2—C1	120.2 (3)	C13—C12—H12	119.5
C3—C2—H2	119.9	C11—C12—H12	119.5
C1—C2—H2	119.9	C14—C13—C12	120.35 (19)
C4—C3—C2	120.5 (3)	C14—C13—H13	119.8
C4—C3—H3	119.8	C12—C13—H13	119.8
C2—C3—H3	119.8	C13—C14—C15	119.51 (18)
C3—C4—C5	120.1 (3)	C13—C14—H14	120.2
C3—C4—H4	120.0	C15—C14—H14	120.2
C5—C4—H4	120.0	C14—C15—C16	120.74 (18)
C4—C5—C6	120.2 (3)	C14—C15—H15	119.6
C4—C5—H5	119.9	C16—C15—H15	119.6
C6—C5—H5	119.9	C15—C16—C11	120.39 (17)
C1—C6—C5	120.1 (3)	C15—C16—H16	119.8
C1—C6—H6	120.0	C11—C16—H16	119.8
C5—C6—H6	120.0	O2—C17—O1	122.01 (16)
C8—C7—C1	179.1 (2)	O2—C17—C18	127.35 (15)
C7—C8—C9	178.1 (2)	O1—C17—C18	110.64 (15)
C10—C9—C8	124.12 (17)	C17—C18—H18A	109.5
C10—C9—H9	117.9	C17—C18—H18B	109.5
C8—C9—H9	117.9	H18A—C18—H18B	109.5
C9—C10—O1	117.69 (15)	C17—C18—H18C	109.5
C9—C10—C11	127.15 (15)	H18A—C18—H18C	109.5
O1—C10—C11	114.96 (14)	H18B—C18—H18C	109.5
C16—C11—C12	118.06 (16)	C17—O1—C10	117.88 (12)

## References

[bb1] Allen, F. H., Kennard, O., Watson, D. G., Brammer, L., Orpen, A. G. & Taylor, R. (1987). *J. Chem. Soc. Perkin Trans. 2*, pp. S1–19.

[bb2] Bruker (2004). *APEX2* and *SAINT* Bruker AXS Inc., Madison, Wisconsin, USA.

[bb3] Chen, Z., Huang, G., Jiang, H., Huang, H. & Pan, X. (2011). *J. Org. Chem.* **76**, 1134–1139. 10.1021/jo1023987 21235260

[bb4] Debergh, J. R., Spivey, K. M. & Ready, J. M. (2008). *J. Am. Chem. Soc.* **130**, 7828–7829. 10.1021/ja803480b PMC2668979 18517202

[bb5] Goossen, L. J. & Paetzold, J. (2004). *Angew. Chem. Int. Ed.* **43**, 1095–1098. 10.1002/anie.200352357 14983443

[bb6] Li, Y., Liu, X., Jiang, H. & Feng, Z. (2010). *Angew. Chem. Int. Ed.* **49**, 3338–3341. 10.1002/anie.201000003 20340147

[bb7] Nakao, Y., Hirata, Y., Tanaka, M. & Hiyama, T. (2008). *Angew. Chem. Int. Ed.* **47**, 385–387. 10.1002/anie.200704095 18000995

[bb8] Sheldrick, G. M. (1996). *SADABS* University of Göttingen, Germany.

[bb9] Sheldrick, G. M. (2008). *Acta Cryst.* A**64**, 112–122. 10.1107/S0108767307043930 18156677

